# Wheat Bran Enhances the Cytotoxicity of Immobilized *Alcaligenes aquatilis* F8 against *Microcystis aeruginosa*

**DOI:** 10.1371/journal.pone.0136429

**Published:** 2015-08-21

**Authors:** Pengfei Sun, Hui Lin, Guan Wang, Ximing Zhang, Qichun Zhang, Yuhua Zhao

**Affiliations:** 1 College of Life Sciences, Zhejiang University, Hangzhou, Zhejiang, China; 2 Institute of Environment Resource and Soil Fertilizer, Zhejiang Academy of Agriculture Science, Hangzhou, Zhejiang, China; 3 College of Environmental and Resource Sciences, Zhejiang University, Hangzhou, Zhejiang, China; University Hospital of the Albert-Ludwigs-University Freiburg, GERMANY

## Abstract

Algicidal bacteria offer a promising option for killing cyanobacteria. Therefore, a new *Alcaligenes aquatilis* strain F8 was isolated to control *Microcystis aeruginosa* in this study. The algicidal activity of strain F8 was dependent on the cell density of *M*. *aeruginosa*, and the maximal algicidal rate of the free bacterium reached 88.45% within 72 h. With a view to its application to the control of *M*. *aeruginosa* in the natural environment, strain F8 was immobilized in sodium alginate beads, but immobilization of the strain decreased its algicidal rate compared to that of the free bacterium. However, addition of wheat bran to the sodium alginate matrix used to immobilize strain F8 not only eliminated the adverse effects of immobilization on the bacteria but also resulted in an 8.83% higher algicidal rate of the immobilized than free bacteria. Exclusion and recovery methods were used to identify key ingredients of wheat bran and gain insight into the mechanism underlying the observed enhancement of algicidal activity. This analysis indicated that certain factors in wheat bran, including vitamins B_1_, B_2_, B_9_, and E were responsible for promoting bacterial growth and thereby improving the algicidal rate of immobilized strain F8. Our findings indicate that wheat bran is able to improve the algicidal efficiency of *A*. *aquatilis* strain F8 for killing *M*. *aeruginosa* and is a good source of not only carbon and nitrogen but also vitamins for bacteria.

## Introduction

Harmful cyanobacteria blooms (HCBs) generated from *Microcystis aeruginosa* commonly occur in aquatic environments worldwide, particularly in China [1, 2]. Previous reports indicated that over 70% of lakes in China are seriously polluted by *M*. *aeruginosa*, which poses a grave threat to not only the regional economic development but also the safety of aquatic animals and humans [3, 4]. Therefore, there is an urgent need to explore feasible approaches for the termination of HCBs.

In recent years, promising strategies for the control of HCBs have been proposed based on the application of algicidal bacteria. These reported algicidal bacteria mainly belong to the genera *Cellvibrio* sp., *Bacillus* sp., *Pseudomonas* sp., and *Ochrobactrum* sp., etc. [5–8]. However, the potential application of these bacteria in HCBs control remains to be studied, and it is still essential to identify novel algicidal bacteria to enrich algicidal bacterial resources for the termination of HCBs, especially those at high cell density. Moreover, specific immobilization techniques are crucial to improve the application of algicidal bacteria in the wild because they provide algicidal bacteria with a stable growth environment, thereby helping them to control HCBs [9–13]. Although immobilization techniques have several advantages, including high reproducibility and high stability, certain problems associated with their use, such as the reduction of bacterial activity, need to be resolved.

In the present study, a new and highly efficient algicidal bacterial strain was isolated, and an immobilization technique was applied to improve its practical use. However, immobilization had the adverse effect of decreasing the algicidal rate of the bacterium. To eliminate this adverse effect, wheat bran was added to the matrix used for immobilization, based on a report of wheat bran inducing the ethanol production of immobilized microbial cells [14]. The addition of wheat bran enhanced the algicidal activity of the immobilized bacterium, and we investigated the underlying mechanism by using ingredient exclusion and recovery methods. Our findings identify a novel method for the termination of *M*. *aeruginosa* using immobilization of algicidal bacteria in combination with the addition of wheat bran to the immobilization matrix, and they also provide new information regarding the role of wheat bran in microbial culture.

## Experiments and Methods

### Strains, cultivation and identification

The *M*. *aeruginosa* FACHB-905 strain used in this study was purchased from the Institute of Hydrobiology, Chinese Academy of Sciences, Wuhan, China. The strain was cultured in Blue-Green Medium 11 (BG11) and transferred once a week to fresh BG11 to ensure that experiments were always conducted with cultures that were in the exponential growth phase [15].

*Alcaligenes aquatilis* strain F8 was originally enrichment-cultured in Luria-Bertani medium [16] from a water sample collected from an artificial lake at Zhejiang University, Hangzhou, China [17]. Thereafter, for verification of algicidal activity, the enriched bacteria were cultured in mineral medium (MM) [17]. In total, 18 strains were isolated after screening and re-screening of the original water sample. Strain F8 (GenBank accession no: KM222493), with a high algicidal activity, was chosen from these isolates. Genomic DNA of strain F8 was extracted using an AxyPrep Bacterial Genomic DNA Miniprep Kit (Axygen Biosciences, Union City, CA, USA). The 16S rDNA was amplified before it was sequenced by Sangon Biotech Co., Ltd. (Shanghai, China). The forward and reverse primers used for 16S rDNA amplification were 27F and1492R. The 16S rDNA sequence obtained from strain F8 was aligned with sequences of related organisms retrieved from the GenBank database using the BLAST algorithm. Sequence alignment was performed using Clustal X software [18], and a neighbor-joining phylogenetic tree was constructed using the Bioedit and Mega 4.0 programs [19].

### Immobilization and de-immobilization of strain F8

Immobilization: Sodium alginate dissolved in MM at a final concentration of 2% was used to immobilize strain F8. Immobilization was conducted in accordance with a previously described method [20]. Thalli of strain F8 were harvested during the exponential growth phase by centrifugation at 7200 *g* for 10 min at 25°C, and then washed twice with potassium phosphate buffer (50 mM, pH 7.0). Thalli (0.33 g, wet weight) were first mixed with 0.25 g of different types of sterilized wheat brans (untreated wheat bran or acid/alkali-treated wheat bran) in 5 mL MM, and then the mixture was added to 20 mL of sodium alginate solution (w/v, 2.5%) to yield a final sodium alginate concentration of 2%. Sodium alginate beads were produced by drop-wise injection of the obtained mixtures into 100 mL of CaCl_2_ solution (w/v, 2%), and then these beads were cross-linked in the same CaCl_2_ solution at 4°C for 24 h before being assayed for algicidal activity against *M*. *aeruginosa*. Thalli of strain F8 immobilized by sodium alginate without any wheat bran was set as the control, which was used to determine the algicidal rate of immobilized strain F8.

De-immobilization: In total, 30 sodium alginate beads were completely dissolved in sodium citrate solution (0.2 M, 5 mL) in a water bath (70°C), and the resulting sodium alginate solutions were used to measure the optical density (OD) at 600 nm (OD_600_) of strain F8 using a 7230G spectrophotometer (Shanghai Precision Instrument Co., Ltd., Shanghai, China). Sodium alginate beads without thalli were similarly dissolved in the sodium citrate solution, which was then used as the zero-adjustment solution for OD_600_ measurements.

### Measurement of algicidal activity

For determining the algicidal activity of the free bacterium against *M*. *aeruginosa*, strain F8 was first incubated in MM at 37°C and 200 rpm for 48 h. Next, 4 mL of strain F8 culture (1 × 10^6^ cells/mL) was mixed with 20 mL of *M*. *aeruginosa* culture (5 × 10^6^, 1 × 10^7^, 5 × 10^7^, 1 × 10^8^, and 5 × 10^8^ cells/mL), and then these mixtures were co-cultured in a light incubator at 25 ± 1°C and 3,000 lx, with a 12-h light/dark cycle for 72 h. Addition of 4 mL of sterile MM to 20 mL of *M*. *aeruginosa* culture (at each of the different cell densities) was used as the control in each case to determine the algicidal rate of 4 mL of blank MM against *M*. *aeruginosa* cultures of different cell density. All cultures were sampled (5 mL) at 12-h intervals for chlorophyll *a* extraction. For determining the algicidal activity of immobilized strain F8 against *M*. *aeruginosa*, 20 mL of *M*. *aeruginosa* culture (1 × 10^7^ cells/mL) was incubated with 120 sodium alginate beads containing immobilized strain F8 with/without different types of wheat under the same conditions described above, and chlorophyll *a* was also extracted from culture samples taken at 12-h intervals.

Chlorophyll *a* was extracted following a modification of our previously reported procedure [17]. *M*. *aeruginosa* cells were collected by centrifugation of a 5-mL culture sample at 7,200 *g* for 5 min; the cells were then suspended in 1 mL of distilled water. The tube containing the cell suspension was incubated in a boiling water bath (100°C) for 3 min, and 4 mL of acetone was added to the cell suspension after it had been left to cool to room temperature in the dark. The mixture was centrifuged as before, after which the OD value at 665 nm (OD_665_) of the upper phase was measured. The chlorophyll *a* content and the corresponding algicidal rate (AR) were calculated as described in Eq 1 [21] and Eq 2, respectively:

$$\rho (\text{Chla})\;=\text{ O}{\text{D}}_{665}\text{nm }\times 13.9$$ (1)

$$\text{AR }=\;\frac{\left(\rho_{Chla1}\;-\;\rho_{Chla2}\right)}{\;\rho_{Chla1}}\times \;100\%$$ (2)

where *ρ*_*(Chla)*_ represents chlorophyll *a* content (μg/L), *ρ*_*Chla1*_ and *ρ*_*Chla2*_ represent the chlorophyll *a* content of the control group and experimental group, respectively.

To observe the algicidal effects of strain F8 against *M*. *aeruginosa*, strain F8 was first cultured in MM for 48 h before centrifugation at 7,200 *g* for 5 min to prepare a cell-free culture filtrate (CCF). Next, 4 mL of CCF was added to 20 mL of *M*. *aeruginosa* cell suspension before the mixture was cultured in a light incubator, as described for co-culture of the free bacterium with *M*. *aeruginosa* earlier in this subsection, and after 72 h of treatment, *M*. *aeruginosa* cells were collected by centrifugation. Transmission electron microscopy (TEM) images of the treated cells were obtained using a JEM-2010 high-resolution electron microscope (JEOL, Japan) with an accelerating voltage of 200 kV.

### Identification of key components of wheat bran

Pre-treatment of wheat bran with diluted acid/alkali was used as an exclusion method to identify the key ingredients of wheat bran responsible for enhancing the algicidal activity of immobilized strain F8. Incubation with diluted acid (w/v, 0.75% H_2_SO_4_, 121°C, 1 h, bran to liquid ratio = 10%) or diluted alkali (w/v, 2.5% H_2_O_2_, pH 11.5, 37°C, 24 h, 200 rpm, bran to liquid ratio = 10%) was used to pretreat the wheat bran [22–24]. The obtained acid/alkali-treated brans were, in turn, washed with distilled water to a neutral pH, dried to constant weight, and milled to a diameter lower than 0.028 mm. Thereafter, the acid/alkali-treated brans were immobilized with strain F8 using sodium alginate according to the aforementioned method, and their respective algicidal activities were measured. Potential key components of wheat bran were identified by comparing the differences in the algicidal activity of the immobilized bacterium resulting from the addition of the two different bran types with the ingredients excluded by the two bran treatments.

### Verification of key components of wheat bran

The method of ingredient recovery, i.e., adding back different ingredients to the acid-treated bran included in the immobilization of strain F8, was employed to verify the key components of wheat bran responsible for enhancing the algicidal activity of the immobilized bacterium. Wheat bran ingredients such as cellulose, starch, and vitamins are removed or destroyed by diluted acid pretreatment, whereas ingredients such as proteins, lipids, some hemicelluloses, and lignin are destroyed by diluted alkali pretreatment [25–32]. Therefore, the three ingredients cellulose, starch, and composite vitamins, were added back to the acid-treated bran used in the immobilization of strain F8, to determine whether the enhancement could be recovered and to identify the key wheat bran ingredient(s). Starch, cellulose, and composite vitamins, or each vitamin, were individually mixed with strain F8 and the acid-treated bran before they were immobilized with sodium alginate. The algicidal rate of immobilized strain F8 in the differently supplemented sodium alginate beads and the corresponding OD_600_ values of de-immobilized strain F8 were assessed after 48 h of co-culture with *M*. *aeruginosa* to analyze the effects of the added ingredients on bacterial growth. In addition, the untreated wheat bran, starch, or cellulose was provided as the sole carbon source in MM to culture strain F8 at 37°C, and 200 rpm for 48 h, followed by measurement of the bacterial growth (OD_600_) and corresponding algicidal rates. All chemicals used in the study were of analytical grade and purchased from Sinopharm Chemical Reagent Co., Ltd. (Shanghai, China).

### Statistical analysis

All assays were repeated at least three times. Data represent three independent experiments and are presented as the mean ± SD. Analysis of variance was performed using SPSS 16.0 software (IBM, Armonk, NY, USA)

## Results and Discussion

### Isolation and identification of strain F8

**Fig 1:**
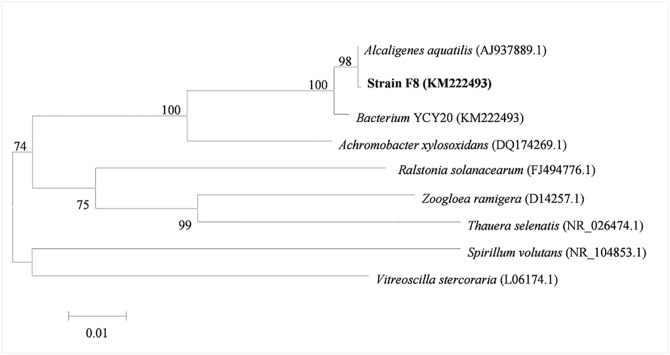
Neighbor-joining phylogenetic tree for *Alcaligenes aquatilis* strain F8 and related species based on 16S rDNA sequences. Bootstrap values (percentages of 1,000 replications) are shown at the branch points. Scale bar = 0.01 substitutions per nucleotide position (evolutionary distance).

The 16S rDNA of isolated strain F8 was PCR-amplified and sequenced, and then the sequence of strain F8 was compared with that of other related bacteria. The results in Fig 1 show that the 16S rDNA sequence of strain F8 had 99% similarity to that of *A*. *aquatilis* and was therefore considered to be *Alcaligenes aquatilis* F8. Although *A*. *denitrificans* has been reported to possess algicidal activity against *M*. *aeruginosa*, this is the first report of algicidal activity in *A*. *aquatilis*, and our results show that these two species had distinct mechanisms to control *M*. *aeruginosa* [33]. Therefore, the discovery is of significance because of the potential to use *A*. *aquatilis* strains to reduce the impact of HCBs caused by *M*. *aeruginosa*.

### Algicidal characteristics of strain F8

**Fig 2:**
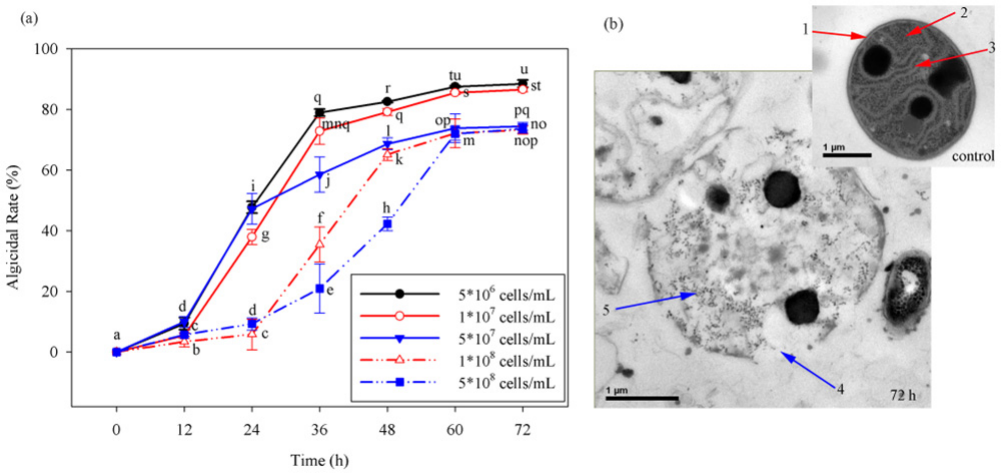
Algicidal characteristics of *Alcaligenes aquatilis* strain F8 against *Microcystis aeruginosa*. (a) Time course of algicidal characteristics of free strain F8. Data represent the mean of three independent experiments, with the SD indicated by an error bar. Different lowercase letters above the columns represent statistically significant differences in algicidal rate among treatment groups at different treatment times for strain F8 against *M*. *aeruginosa* (*p* < 0.05). (b) Transmission electron microscopy images of an untreated *M*. *aeruginosa* cell (shown as control) and a *M*. *aeruginosa* cell treated with strain F8 cell-free culture filtrate for 72 h (shown as treatment). Arrows indicate the following: 1: cell membrane; 2: photosynthetic lamellae; 3: cyanelle; 4: damaged cell membrane; 5: disordered cyanelle without photosynthetic lamellae to adhere to.

The results demonstrate cell density-dependent algicidal activity of free strain F8 against *M*. *aeruginosa* (Fig 2a). The algicidal rate reached 82.56% within 48 h, with an initial cell density of *M*. *aeruginosa* reaching 5 × 10^6^ cells/mL, and the maximal algicidal rate reached 88.45% within 72 h. For an initial *M*. *aeruginosa* cell density of 1 × 10^7^ cells/mL, the algicidal rate was decreased by approximately 2–3% compared with the algicidal rate for an initial cell density of 5 × 10^6^ cells/mL at each time point. When the initial cell density was increased to 5 × 10^7^ cells/mL, the values for both maximal algicidal rate and algicidal velocity were decreased relative to the corresponding values for initial cell densities of 5 × 10^6^ and 1 × 10^7^ cells/mL, particularly between 36 h and 72 h (*p* < 0.05), with algicidal rates of 58.56%, 68.68%, 73.79%, and 74.41%, respectively. Initial *M*. *aeruginosa* cell densities of 1 × 10^8^ and 5 × 10^8^ cells/mL resulted in similar algicidal rates (of <10%) within 24 h (*p* > 0.05), with an increasing trend observed with time from 24 h to 60 h. The maximal algicidal rates reached approximately 73% at 60 h and remained constant thereafter (73.59%). These results indicate the superiority of strain F8 in the control of *M*. *aeruginosa* compared to other reported algicidal bacteria. For instance, the maximal algicidal efficiency reported for *Ochrobactrum* sp. against *M*. *aeruginosa* (5×10^6^ cells/mL) is 80% [8], and *Pseudomonas fluorescens* SK09 requires 5 days to lyse approximately 90% of a *Stephanodiscus hantzschii* culture (5 × 10^6^ cells/mL) [34]. Therefore, the discovery of strain F8 offers a new algicidal bacterium resource to eliminate HCBs generated by *M*. *aeruginosa*, even those at high cell density.

The algicidal effect of strain F8 against *M*. *aeruginosa* was observed by TEM. As the TEM images in Fig 2b show, the cell membrane (arrow 1) of normal *M*. *aeruginosa* cells (control) was intact, and many cyanelles (arrow 2) coherently adhered to photosynthetic lamellae (arrow 3), which were ordered within the cells. When *M*. *aeruginosa* cells were co-cultivated with CCF of strain F8 for 72 h, their cellular morphology changed considerably. TEM imaging at 72 h shows that the cell membrane was damaged (arrow 4), photosynthetic lamellae had disappeared (arrow 5), and cyanelles were disordered because there were no photosynthetic lamellae for them to adhere to. Taken together, the results in Fig 2b are indicative of the algicidal properties of strain F8 against *M*. *aeruginosa*.

### Wheat bran enhances the algicidal activity of immobilized strain F8

**Fig 3:**
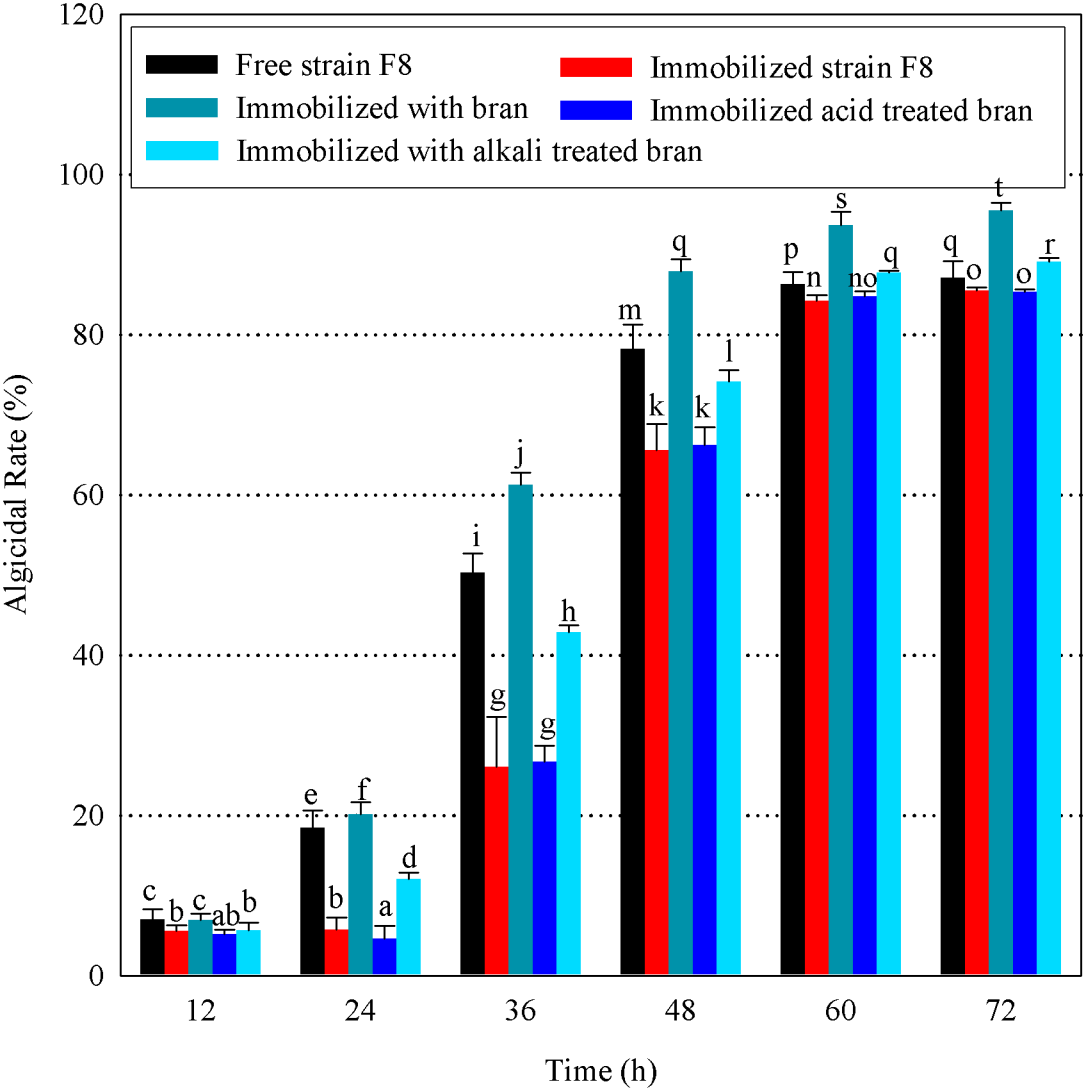
Algicidal characteristics of strain F8 under different immobilization conditions. Data represent the mean of three independent experiments with the SD indicated by an error bar. Different lowercase letters above the columns represent statistically significant differences in algicidal rate among treatment groups at different treatment times for strain F8 against *M*. *aeruginosa* (*p* < 0.05).

With a view towards applying strain F8 in the natural environment, an immobilization technique was used to support the bacterium and potentially enhance its algicidal activity. However, when the algicidal activity of immobilized strain F8 was compared with that of the free bacterium, immobilization was found to significantly reduce the algicidal rate, especially at the early stages (24, 36, and 48 h) in the algicidal process (*p* <0.05, Fig 3). Interestingly, mixing strain F8 with wheat bran prior to immobilization in sodium alginate beads remarkably enhanced the algicidal rate of the immobilized strain compared to that of the free bacterium (*p* <0.05); the maximal algicidal rates measured at 72 h of co-culture with *M*. *aeruginosa* cells were 87.06% (free strain F8), 85.46% (immobilized strain F8), and 95.49% (strain F8 immobilized with wheat bran). Thus, wheat bran, a common agricultural by-product, can be used to improve the algicidal efficiency of immobilized strain F8 against *M*. *aeruginosa*. This result is similar to those previously reported by Kang et al. and Patil et al. [35, 36].

### Mechanism of algicidal enhancement by wheat bran

#### Identification of key components of wheat bran

The results in Fig 3 show that the observed enhancement of the algicidal rate by wheat bran disappeared after the wheat bran was treated with diluted acid. The algicidal rate of strain F8 immobilized with acid-treated bran was nearly equal to that of immobilized strain F8 alone (*p* > 0.05), however, when wheat bran was treated with diluted alkali, the enhancement was only slightly decreased. We therefore deduced that the acid treatment process caused the destruction of key ingredients in wheat bran that were required for increasing the algicidal rate of immobilized strain F8.

The complex composition of wheat bran, which is rich in cellulose, starch, and vitamins, posed a challenge for the identification of the key components responsible for enhancing the algicidal rate [37, 38]. Therefore, an exclusion strategy was used to identify potential key ingredients. Diluted acid treatment primarily removes components such as starch, cellulose, and vitamins from bran, whereas other hemicelluloses, lignin, proteins, and lipids are mainly removed by diluted alkali treatment [24]. Based on the algicidal rates observed after addition of diluted acid- or alkali-treated bran to the sodium alginate matrix used to immobilize strain F8, we concluded that components such as proteins and lipids were not the key ingredients; however, further experiments were needed to determine whether ingredients such as starch, cellulosic substances, and vitamins were the key ingredients.

#### Verification of potential key components of wheat bran

Wheat bran components such as starch, cellulosic substances, and vitamins could not be directly determined as key ingredients by the initial exclusion experiments; therefore, they were further evaluated by ingredient recovery and sole-carbon-source experiments.

**Fig 4:**
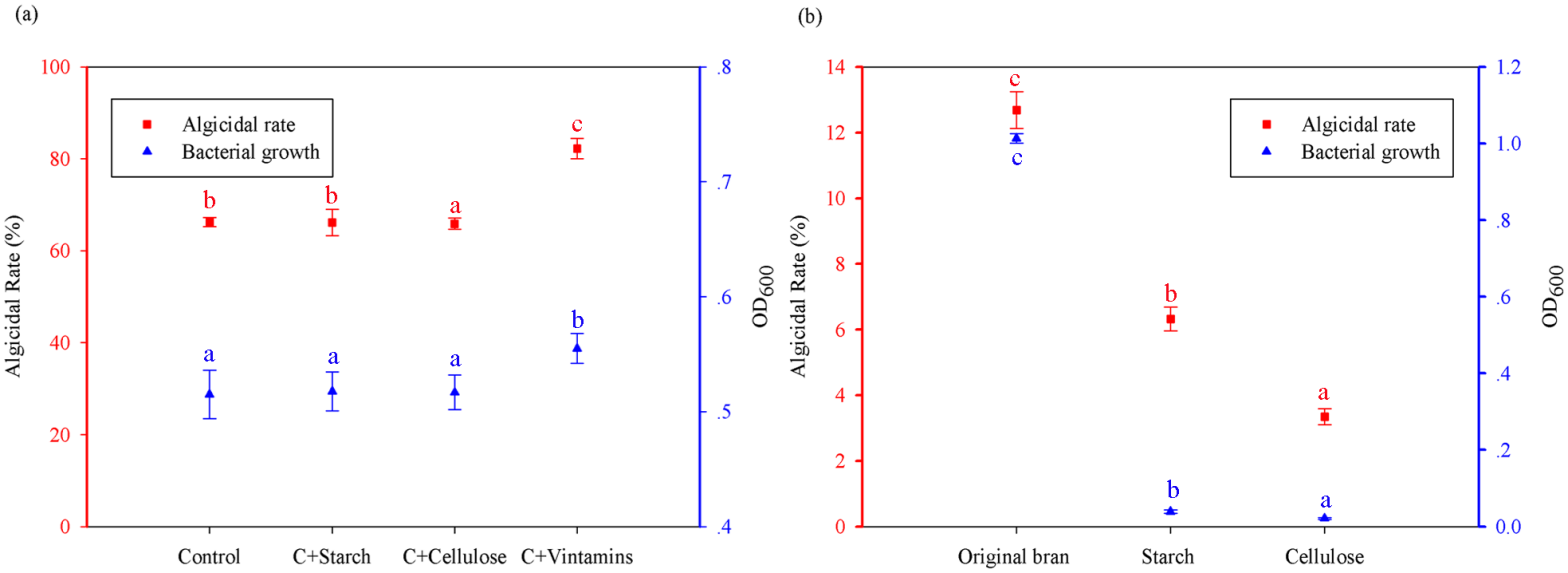
Effects of added-back ingredients on the algicidal rate and growth of *Alcaligenes aquatilis* strain F8 immobilized with acid-treated bran. (a) Ingredients added back to acid-treated bran during the immobilization of strain F8. Control (‘C’): no addition; ‘C + Starch’, ‘C + Cellulose’, and ‘C + Vitamins’ represent the addition of starch, cellulose, or vitamins, respectively. (b) Ingredients added back as sole carbon sources. Data represent the mean of three independent experiments with the SD indicated by an error bar. Different lowercase letters above the columns represent statistically significant differences in growth of strain F8 under different treatments, and in algicidal rate among treatment groups at different treatment times for strain F8 against *Microcystis aeruginosa* (*p* < 0.05).

The average contents of the main ingredients of wheat bran, as reported from previous studies [37, 38] are shown in Table 1, and the corresponding amounts used in ingredient recovery experiments, i.e., added back to acid-treated bran during immobilization of strain F8 in sodium alginate beads, are shown in Table 2. The results in Fig 4a reveal that the algicidal rate in the composite vitamin adding back group (C + vitamins, 82.24%) was markedly higher than that in the control group (66.26%), starch adding back group (C + starch, 66.16%) and cellulose adding back group (C + cellulose, 65.89%) (*p* < 0.05). These results indicate that there was no enhancement of algicidal rate by adding back starch or cellulose, whereas the algicidal rate was significantly recovered (*p* < 0.05) by adding back composite vitamins to acid-treated bran during the immobilization of strain F8. The OD_600_ values of strain F8 in sodium alginate beads with different added ingredients were also measured after 48 h in culture. The results in Fig 4a indicate no significant difference (*p* > 0.05) in the growth of strain F8 in the control group (OD_600_ = 0.5150), starch adding back group (OD_600_ = 0.5176), or cellulose adding back group (OD_600_ = 0.5168), whereas the growth of strain F8 in sodium alginate beads was significantly enhanced (*p* < 0.05) by adding back vitamins (OD_600_ = 0.5550). Therefore, the addition of composite vitamins promoted the growth of strain F8 and enhanced the algicidal activity of strain F8 immobilized with acid-treated bran.

**Table 1:** Composition and average content of 100 g of wheat bran.

Composition	Content	Composition	Content
Protein^a^	15	B_3_ ^b^	200
Starch^a^	50	B_6_ ^b^	10
Cellulose^a^	30	B_9_ ^b^	2
Lipid^a^	5	E^b^	10
B_1_ ^b^	10	Biotin^b^	10
B_2_ ^b^	5		

^a^unit: g

^b^unit: mg

**Table 2:** Average amounts of different wheat bran components added back to the acid-treated bran included in the immobilization of *Alcaligenes aquatilis* strain F8.

	Cellulose		CVs^b^				
Starch^a^	Xylan^a^	CMC^a^	B_1_+…+Biotin	B_1_ ^b^	B_2_ ^b^	B_3_ ^b^	B_6_ ^b^
0.250	0.070	0.035	0.618	0.025	0.013	0.500	0.025
B_9_ ^b^	E^b^	Biotin^b^					
0.005	0.025	0.025					

CMC: sodium carboxymethylcellulose

CVs: composite vitamins

Cellulose consisted of xylan and CMC; CVs were a mixture of B_1_, B_2_, B_3_, B_6_, B_9_, E, and biotin.

^a^unit: g

^b^unit: mg

The observation that neither starch nor cellulose had any effect on the growth or the algicidal rate of strain F8 was further verified by sole-carbon-source analysis. In this analysis, wheat bran, starch, and cellulose were individually used as the sole carbon source in MM culture of strain F8. Strain F8 grew well in MM with wheat bran as the sole carbon source (OD_600_ = 1.014), but the corresponding algicidal rate was quite low (12.69%; Fig 4b), which implies that wheat bran can be utilized by these bacteria as a carbon source for growth, but it is not optimal for *M*. *aeruginosa* lysis. The growth of strain F8 in MM containing starch or cellulose as the sole carbon source was comparatively poorer (OD_600_ values of 0.039 and 0.02, respectively, *p* < 0.05), and the corresponding algicidal rates were similarly lower (6.32% and 3.34%, respectively, *p* < 0.05). These sole-carbon-source experiments revealed that starch and cellulose are hardly utilized as carbon sources for growth by strain F8 and cannot be used to enhance its algicidal activity.

Overall, our results suggest that wheat bran is not an optimal carbon source for strain F8 in the control of *M*. *aeruginosa*, but it is useful for enhancing the algicidal rate because vitamins in wheat bran promote the growth of the strain.

#### Effects of individual vitamins on the algicidal rate of immobilized strain F8

**Fig 5:**
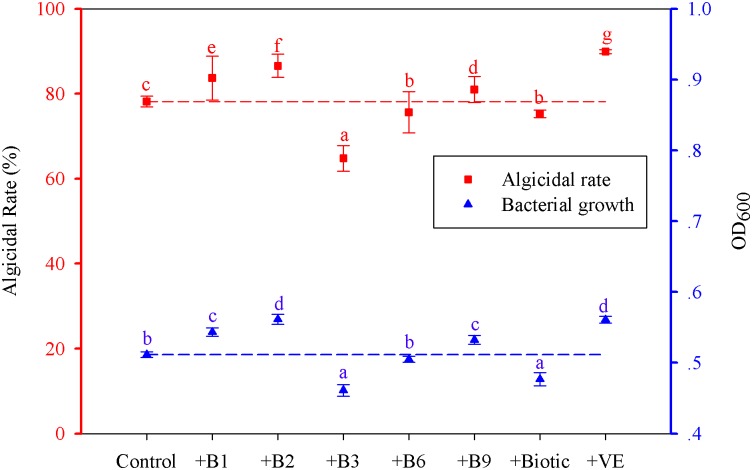
Effects of individual vitamins on the algicidal rate and growth of *Alcaligenes aquatilis* strain F8 immobilized with acid-treated bran. ‘Control’: strain F8 immobilized with acid-treated bran; ‘+B_1_, +B_2_,…+E’: adding back of the respective individual vitamins to acid-treated bran during the immobilization of strain F8. Data represent the mean of three independent experiments with the SD indicated by an error bar. Red and blue dotted lines represent the control values of algicidal rate and bacterial growth (OD_600_), respectively. Different lowercase letters above the columns represent statistically significant differences in growth of strain F8 under different treatments, and in the algicidal rate among treatment groups at different treatment times for strain F8 against *Microcystis aeruginosa* (*p* < 0.05).

To determine which vitamin in wheat bran contributed to the enhancement of algicidal activity, more detailed analyses were conducted. The results show that the control (strain F8 immobilized with acid-treated bran) reached an OD_600_ value of 0.5115 and the corresponding algicidal rate against *M*. *aeruginosa* of 78.20% within 48 h (Fig 5). The OD_600_ values of the control with addition of the individual vitamins (B_1_, B_2_, B_3_, B_6_, folic acid (B_9_), biotin, and E) were 0.5435, 0.5615, 0.4612, 0.5050, 0.5325, 0.4768, and 0.5610, respectively; the corresponding algicidal rates were 83.74%, 86.60%, 64.81%, 75.65%, 81.05%, 75.24%, and 89.95%, respectively. Thus, vitamins B_1_, B_2_, B_9_, and E markedly promoted the growth of strain F8 (*p* < 0.05), whereas the other vitamins inhibited its growth. Furthermore, the growth effect of each vitamin echoed its effect on the algicidal rate; vitamins B_1_, B_2_, B_9_, and E significantly enhanced the algicidal rate of immobilized strain F8 against *M*. *aeruginosa* (*p* < 0.05), with maximal enhancement by vitamin E followed by vitamins B_2_, B_1_, and B_9_, which was the same pattern as observed for the growth of strain F8.

Collectively, our results indicate that wheat bran enhances the algicidal rate of immobilized strain F8 as vitamins B_1_, B_2_, B_9_, and E promote the growth of the bacteria. Importantly, wheat bran is an inexpensive and common agricultural by-product that appears to be suitable as an auxiliary material to improve the algicidal efficiency of immobilized strain F8 in the elimination of *M*. *aeruginosa*. Furthermore, this study shows that wheat bran is a good source of not only carbon and nitrogen but also vitamins for bacteria.

## Conclusions

The present study indicates that *A*. *aquatilis* strain F8 is a novel and effective microbe for eliminating *M*. *aeruginosa*. The maximal algicidal efficiency reached 88.45% within 72 h. Wheat bran eliminated the adverse impact of immobilization on the algicidal rate, and even increased the algicidal activity to 8.83% higher than that of the free bacterium. Vitamins B_1_, B_2_, B_9_, and E were the key components of wheat bran responsible for the enhanced algicidal rate achieved through promotion of the growth of strain F8. These findings indicate that wheat bran can be used to improve algicidal efficiency for elimination of *M*. *aeruginosa*.
